# Spatiotemporal Imaging of Zinc Ions in Zebrafish Live Brain Tissue Enabled by Fluorescent Bionanoprobes

**DOI:** 10.3390/molecules28052260

**Published:** 2023-02-28

**Authors:** Romana Jarosova, Sarah K. Woolfolk, Noraida Martinez-Rivera, Mathew W. Jaeschke, Eduardo Rosa-Molinar, Candan Tamerler, Michael A. Johnson

**Affiliations:** 1 Department of Chemistry and R.N. Adams Institute for Bioanalytical Chemistry, University of Kansas, Lawrence, KS 66045, USA; 2 UNESCO Laboratory of Environmental Electrochemistry, Department of Analytical Chemistry, Charles University, 12843 Prague 2, Czech Republic; 3 Institute for Bioengineering Research, University of Kansas, Lawrence, KS 66045, USA; 4 Bioengineering Program, University of Kansas, Lawrence, KS 66045, USA; 5 Microscopy and Analytical Imaging Research Resource Core Laboratory, University of Kansas, Lawrence, KS 66045, USA; 6 Department of Mechanical Engineering, University of Kansas, Lawrence, KS 66045, USA; 7 Department of Pharmacology & Toxicology, University of Kansas, Lawrence, KS 66045, USA

**Keywords:** zinc, gold, nanoparticles, zebrafish, two-photon excitation imaging, fluorescence

## Abstract

The zebrafish is a powerful model organism to study the mechanisms governing transition metal ions within whole brain tissue. Zinc is one of the most abundant metal ions in the brain, playing a critical pathophysiological role in neurodegenerative diseases. The homeostasis of free, ionic zinc (Zn^2+^) is a key intersection point in many of these diseases, including Alzheimer’s disease and Parkinson’s disease. A Zn^2+^ imbalance can eventuate several disturbances that may lead to the development of neurodegenerative changes. Therefore, compact, reliable approaches that allow the optical detection of Zn^2+^ across the whole brain would contribute to our current understanding of the mechanisms that underlie neurological disease pathology. We developed an engineered fluorescence protein-based nanoprobe that can spatially and temporally resolve Zn^2+^ in living zebrafish brain tissue. The self-assembled engineered fluorescence protein on gold nanoparticles was shown to be confined to defined locations within the brain tissue, enabling site specific studies, compared to fluorescent protein-based molecular tools, which diffuse throughout the brain tissue. Two-photon excitation microscopy confirmed the physical and photometrical stability of these nanoprobes in living zebrafish (*Danio rerio*) brain tissue, while the addition of Zn^2+^ quenched the nanoprobe fluorescence. Combining orthogonal sensing methods with our engineered nanoprobes will enable the study of imbalances in homeostatic Zn^2+^ regulation. The proposed bionanoprobe system offers a versatile platform to couple metal ion specific linkers and contribute to the understanding of neurological diseases.

## 1. Introduction

Zinc is one of the most abundant transition metals in the brain, second only to iron [1]. In its free, ionic form (Zn^2+^) it plays a critical role in various neurological diseases [2,3], influencing neuronal function [4,5] by guiding neuronal communication, memory formation, and sensory processing [6]. Zinc exists in bound and unbound forms, and levels as free ions may rapidly fluctuate. For example, about 80–90% of zinc exists as an integral part of proteins while the remaining 10–20% exists in its free form (Zn^2+^) and is stored, in part, in synaptic vesicles [1]. This vesicular Zn^2+^ undergoes Ca^2+^ dependent exocytosis from subsets of glutamatergic neurons in the hippocampus, amygdala, striatum, and thalamus [7,8,9,10,11]. This release of Zn^2+^, estimated to be present in vesicles at low mM concentrations, transiently increases synaptic (100–300 µM) and extracellular (10–20 µM) Zn^2+^ levels [7]. Given its important physiological roles in neuronal health and function, understanding the factors affecting zinc ion homeostasis in living brain tissues could contribute immensely to our current understanding of the mechanisms that underlie neurological disease pathology [12,13,14,15,16].

The general categories for measuring Zn^2+^ levels have commonly included genetically encoded Zn^2+^ indicators (GEZIs) and small molecule sensors (reviewed by Pratt et al. 2021) [17]. A wide range of genetically encoded Zn^2+^ sensors have been developed to measure Zn^2+^ levels in the cytosol [18] and various organelles, including the endoplasmic reticulum [18], Golgi apparatus [18], mitochondrial matrix [19], mitochondrial intermembrane space [20], lysosomes [21,22], and secretory vesicles [23]. Additionally, recent work has demonstrated the use of genetically encoded sensors for detecting Zn^2+^ on the extracellular surface in cultured cell preparations [2]. However, the optical detection of free metal ion distribution in biological systems, especially in the brain, has proven challenging due to spatial and temporal limitations.

In the past few decades, the use of metal ion sensors that incorporate small organic molecules, peptides, and proteins have been instrumental in revealing zinc ion changes [17,24,25]. The co-existence of multiple metal ion types in different quantities and in complex biological environments requires the development of additional visualization methods. The ability to tune these sensors by the choice of fluorophore and chelator offers distinct advantages. With the limitation of several organic or chromogenic dyes absorbing light, fluorescent based approaches have attracted increasing interest for monitoring transition metals in biological systems. Biochemical molecular constructs as well as synthetic probes have been utilized for the fluorescent imaging of transition metal ions. Molecular sensors were developed to measure intracellular free Zn^2+^ based on carbonic anhydrase conjugated to small molecules, such as AlexaFluor, as the fluorophore [26]. With the advent of these molecular sensors and fluorescence approaches, there has been a steady increase in the variety of fluorescent Zn^2+^ sensors. However, although molecular probe sensors are easy to inject, they diffuse instantly. Additional potential roadblocks to using them in complex living brain tissues include decreased bioavailability due to the inability to cross the blood–brain barrier and breakdown by metabolic and catabolic enzymes. These molecular tools also are not well-suited for obtaining measurements in spatially confined spots within the brain. Furthermore, several of these studies were conducted in primary neuron cultures and brain slices of mice or other small animals.

Owing to their unique optical properties, colloidal metal nanoparticles have been widely used for a broad range of metal ion detection methods [27,28]. Profuse fluorescent nanoprobes have been developed to detect metal ions for medical diagnostics, biomedical imaging, and environmental monitoring. Gold nanoparticles (AuNPs) are among the most used nanomaterials in the biomedical fields due to their unique optical and electronic properties, excellent chemical stability, and biological compatibility. Numerous synthesis and functionalization methods have been used for AuNPs to construct fluorophore and quencher-based sensing systems for several metal ions, including Zn^2+^ [29,30,31,32,33,34]. The surface functionalization of metal nanoparticles is especially attractive as they have the potential to be expanded into multimodal imaging applications. In biological systems, proteins are central in guiding molecular recognition to self-assembly with molecular scale precision. Inspired by biological processes, we and other groups have selected inorganic specific peptides using biocombinatorial techniques, and enhanced their binding kinetics and self-assembly properties using computational and experimental techniques [35,36,37,38]. These peptides allow the biofunctionalization of nanoparticle surfaces with high affinity and selectivity, with the ability to display additional bioactive peptides and/or proteins to target a desired functionality. We recently explored the formation of self-assembled hybrid metallic nano-architectures composed of gold and silver nanoparticles with green fluorescent proteins as bimodal imaging probes [39]. We employed metal-binding, peptide-based assembly to self-assemble green fluorescence protein onto metallic nanoparticles, i.e., Au and Ag [40,41,42]. Such systems allow monitoring using both plasmonic and fluorescent signatures. We recently expanded these approaches to design fluorescent proteins, GFP and DsRed proteins, as reporter molecules for rapid quenching in the presence of inorganic ions including Cu^2+^ [39]. These reporter proteins, designed as chimeric fusion systems, self-assemble on metallic nanoparticles using in vitro systems. Despite several advances in metallic nanoprobes, functionalized with synthetic or biomolecular reporters, the detection of metal ions with colloidal metal NP-based fluorescence in living cells or brain tissues has remained limited.

**Figure 1:**
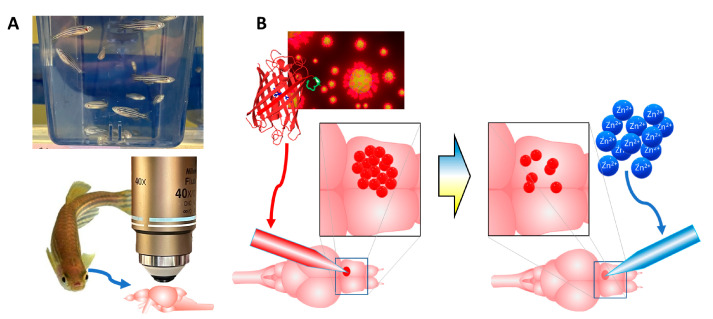
Schematic diagram of the overall experimental paradigm. (**A**), Live, whole brains from zebrafish were harvested for microscopy imaging. (**B**), Bionanoprobes (red balls) were delivered to specific locations in the brain with a picospritzer. Next, exogenous Zn^2+^ ions (blue balls) were delivered though a separate capillary to induce quenching. The delivery of the bionanoprobes and Zn^2+^ was imaged with conventional and 2PE fluorescence microscopy. Credit for image of individual zebrafish: David Dohnal/Shutterstock.com.

Here, we investigated a self-assembled bionanoprobe that is composed of an engineered red fluorescent protein and gold nanoparticles as a molecular tool enabling the detection of Zn^2+^ in living zebrafish brain tissue (see Figure 1). We showed that the ability of these bionanoprobes to be quenched is preserved in live, whole brains removed from zebrafish, and provides a high degree of spatial and temporal resolution for the detection of ions. The use of whole brains ex vivo from this rapidly emerging research model holds key advantages. First, the whole brains are small enough to be kept viable and functional in a superfusion chamber. Because the brain is kept whole, all the neural pathways are preserved and functional. Additionally, the small size of the zebrafish brains facilitates light microscopy imaging measurements and is especially amenable to two-photon excitation (2PE) microscopy, which can image within the opaque tissue of adult brains. For these reasons, this model organism is ideal for the study of free biometals in homeostatic regulation and for understanding neurologically relevant disease models. To our knowledge, the optical detection of free metal ions in a living whole brain with gold nanoparticles, functionalized with fluorescent proteins, has not been published in the peer reviewed literature. The use of our bionanoprobes offers a unique, enabling platform with its versatile self-assembled design to target different metal ions by engineering different specific moieties into the protein as well as nanoparticle assemblies.

## 2. Results and Discussion

**Figure 2:**
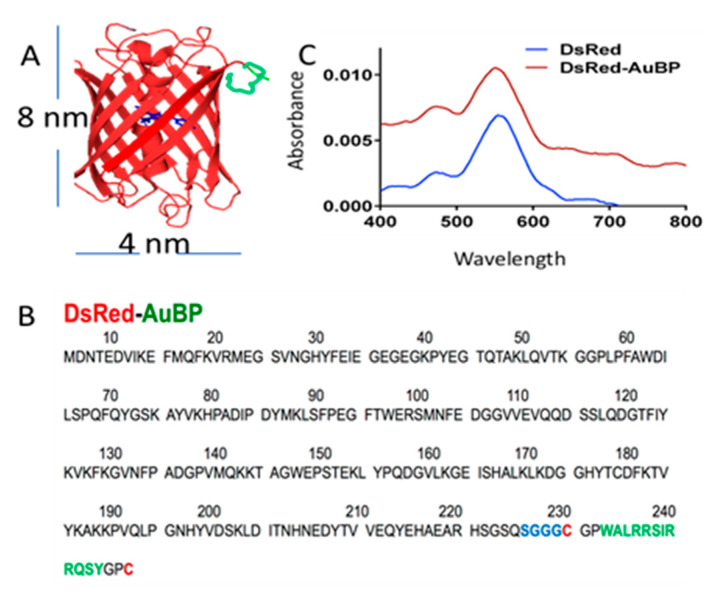
DsRed protein engineered with Au binding peptide. (**A**), The peptide insertion includes Au binding peptide (Green), linker (blue), and conformational domains (red and purple). (**B**), Insertion locations of DsRed-AuBP. (**C**), Fluorescence spectra showing that the properties of DsRed are conserved after the insertion of AuBP.

To generate the self-assembled nanoprobe system, we used peptide motifs that were identified and studied in detail for their selective metal binding property and with high affinity to gold surfaces and nanoparticles [35,36,37,38] (Figure 2). The genetic incorporation of gold binding peptide (Figure 2A,B) as a tag into the DsRed fluorescent protein (λ_ex_ = 545 nm, λ_em_ = 573 nm) enabled the use of the self-assembled, fluorescent gold nanoparticles as bimodal imaging nanoprobes [39] (Figure 2C). The fabrication and use of these probes were carried out using proper safety and disposal measures. The resulting nanoprobe was explored as a potential metal ion-monitoring tool in living zebrafish brain tissue due to its experimental advantages and the high sequence homology of zebrafish to mammals [43,44,45].

**Figure 3:**
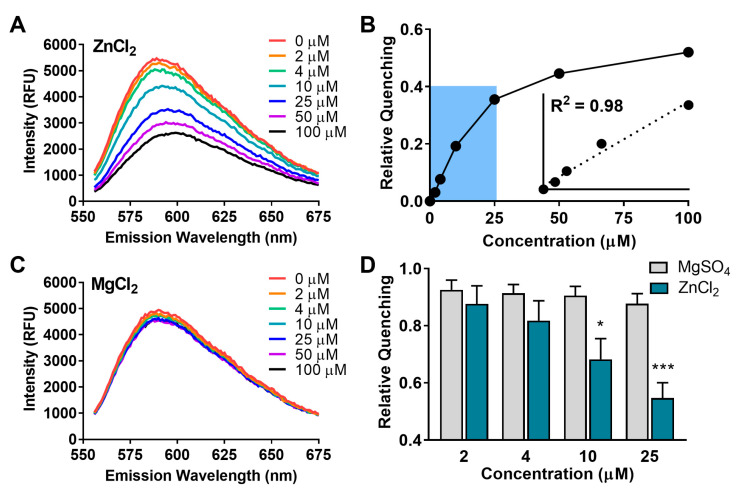
Nanoprobe response to metals in vitro. (**A**), Zn^2+^ response curves (0 to 100 μM) and (**B**), representative calibration plot (inset from shaded region, 0 to 25 μM). (**C**), Mg^2+^ response curves (0 to 100 μM) and (**D**), Quenching vs. concentration of added MgSO_4_ and ZnCl_2_. Bars on graph indicate mean ± SEM. Statistics: * *p* < 0.05 vs. MgSO_4_, *** *p* < 0.001 vs. MgSO_4_, (two-way ANOVA with Sidak post hoc, *n* = 4).

We first ran in vitro quenching assays using Zn metal ions with nanoprobes that were composed of 15 nm-diameter gold nanoparticles functionalized with the fusion DsRed-AuBP protein (Figure 3A–D). Figure 3B shows that the dynamic linear range of the probes with respect to Zn^2+^ extends to about 25 μM (R^2^ = 0.98). Given that the expected transient brain concentrations of Zn^2+^ (10–20 μM) fall within the linear dynamic range, our nanoprobes seem well-suited for measurements of extracellular metal release. We next tested the probes for selectivity for Mg^2+^ due to its presence in the extracellular fluid in the brain. Analysis by two-way ANOVA revealed the significant overall main effects of metal ion identity (*p* = 0.007, F [3,48] = 6.76) and concentration (*p* < 0.0001, F [3,48] = 12.15) on quenching. Further Sidak post hoc testing revealed the high selectivity of the probes for Zn^2+^ over Mg^2+^ (*p* < 0.05 at 10 μM and *p* < 0.001 at 25 μM, two-way ANOVA)(Figure 3D). The selectivity of our nanoprobes for Zn^2+^ at 10 μM was critical to achieve because this concentration approximates the Zn^2+^ levels that occur after release events in living brain tissue. We also note that there could be an interference on the sensing activity with the presence of biologically relevant metal ions, such as sodium and potassium. Building upon the promise of these results, nanoprobes should be investigated in the presence of different metal ions. Nevertheless, our results provide a promising concentration-dependent quenching effect on the fluorescence protein displayed on the nanoprobes.

**Figure 4:**
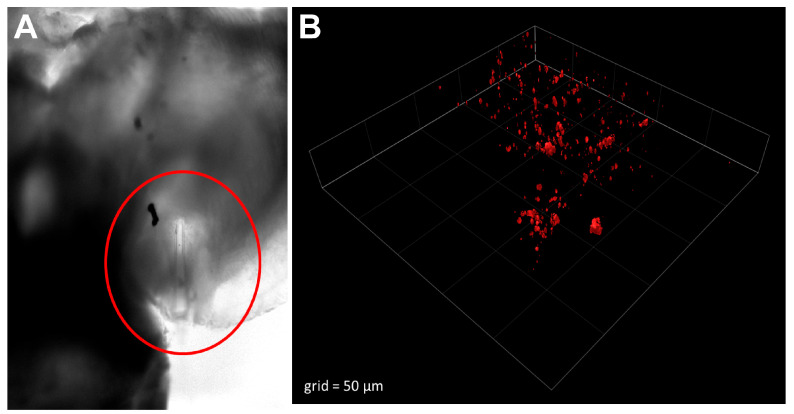
Bionanoprobes were precisely injected into living zebrafish brain. (**A**), Brightfield image of injection capillary (circled) inserted into the hypothalamus. (**B**), Three-dimensional rendering of a 2PE microscopy image of bionanoprobes.

Our initial biological evaluation of these bionanoprobes consisted of direct injection into the hypothalamus of a live zebrafish brain and then imaging with 2PE microscopy. This approach enabled us to observe and optically manipulate the nanoparticle-based systems at the targeted sites with high spatial precision. Shown in Figure 4A is a brightfield image of a typical pulled capillary used for injection, inserted into the brain with a micromanipulator. A higher magnification image of the brain with 2PE microscopy demonstrated that the injected fluorescent bionanoprobes were easily imaged with this method (Figure 4B).

**Figure 5:**
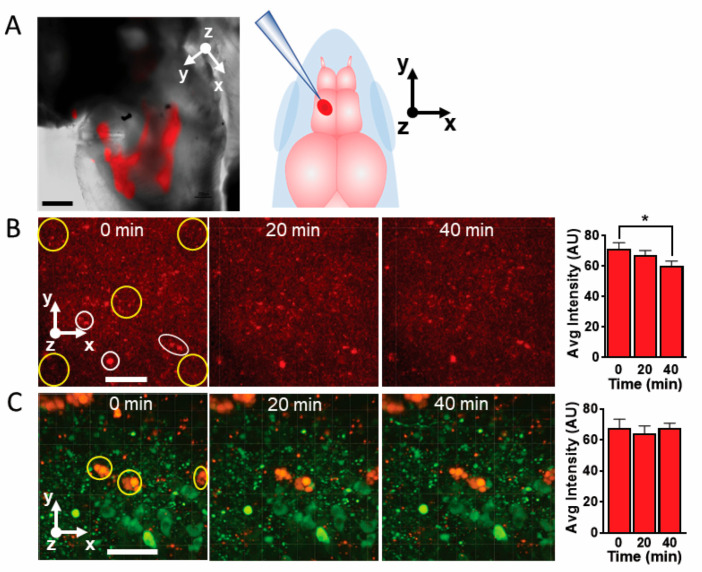
Nanoprobes remain immobile while free DsRed protein molecules tend to diffuse. (**A**), DsRed-AuBP functionalized nanoparticles, 15 nm diameter injected in the ventral diencephalon area of a viable zebrafish brain. Image obtained by overlaying fluorescence on brightfield images. The diagram on the right indicates the approximate location and orientation of spritzing. (**B**), Three-dimensional 2PE image of injected DsRed protein in living zebrafish brain. Sampled regions (yellow circles) and reference points (white circles and ellipse) are depicted at 0 min. The bar graph on the right indicates the average fluorescence intensity inside the yellow circles. Scale bar = 50 µm. * *p* = 0.0091 (two-tailed *t*-test, n = 5 regions sampled per time point). *Z*-axis slice thickness = 80 µm. Orientation: *x*-axis, caudal; *y*-axis, lateral; *z*-axis, ventral. (**C**), DsRed-BP-AuNPs, 15 nm diameter, were picospritzed in live zebrafish brain and imaged with 3D 2PE microscopy. The spatial relationships between the GFP-labeled dopamine neurons (green) and nanoprobes (red) are unchanged. The bar graph on the right indicates the average fluorescence intensities of the regions enclosed in yellow circles and ellipses measured over time. Orientation: *x*-axis, caudal; *y*-axis, lateral; *z*-axis, ventral. 3D images in B and C were captured on a Zeiss Axio Examiner.Z1.

A useful characteristic of our nanoprobes is that they remain stationary in brain tissue while free, unbound DsRed protein tends to diffuse. To illustrate this point, we injected unbound DsRed protein molecules into living zebrafish whole brain maintained in a superfusion chamber and imaged it with 2PE microscopy (injection location shown in Figure 5A). The epifluorescence image of injected nanoprobes overlayed on a brightfield image of a zebrafish telencephalon reveals that these nanoprobes tended to disperse over a wide region of the brain immediately following injection.

The free DsRed protein molecules tended to disperse throughout the brain (Figure 5B). Larger aggregates formed and remained stationary within the brain. To determine if smaller aggregates and individual protein molecules moved, we chose several larger, stationary aggregates as anchor points, and measured how the fluorescence intensity changes over time at defined regions of interest within a selected panel. Over the course of 40 min, the fluorescence intensity within the regions of interest decreased significantly, suggesting that free DsRed protein molecules and smaller aggregates tend to diffuse throughout the tissue.

To determine if nanoparticle clusters move in relation to individual neurons, we also injected nanoprobes into the diencephalon of a living brain from a *th2:gfp* zebrafish. These zebrafish express green fluorescent protein in the dopaminergic neurons. We then imaged the brain and the adjacent fluorescent dopaminergic neurons with 2PE microscopy at 0, 20, and 40 min after injection (Figure 5C). Using this approach, the color differences between the DsRed nanosensors and the GFP allows us to simultaneously observe the nanoprobes and neurons within the same image over time.

As shown in Figure 5C, the bionanoprobe clusters do not appear to move significantly in relation to the GFP-labeled neurons. Another possible barrier to using our nanoprobes to sense Zn^2+^ or other analytes would be the potential degradation by photobleaching or protease activity in the brain, a common issue observed using small molecules, organic dyes, or quantum dots [46]. However, observation of the images indicates that the nanoprobes continued to fluoresce without noticeable photobleaching for at least 40 min. Thus, we conclude that our bionanoprobe system is stationary and more stable compared to peptide fluorophores and is well suited for applications in brain tissue.

Interestingly, careful inspection of the images in Figure 5C reveals that some of the smaller nanoprobe clusters or individual probes slightly migrate or disappear over time. While we do not know the precise cause of these disappearances, we speculate that they may move slightly in the extracellular space or undergo endocytosis [47]. Given that divalent metal ions, including Zn^2+^, are present in the endosomes/lysosomes as well as in the cytosol, quenching may occur upon nanoparticle uptake [48].

**Figure 6:**
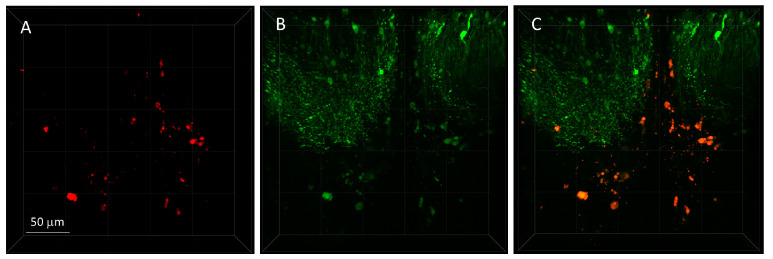
Fluorescent image of bionanoprobe clusters. (**A**) Fluorescence of DsRed protein on nanoprobes measured at 950 nm. (**B**) Fluorescence of gold nanoparticles measured at 850 nm. (**C**) Combined. Images collected on a 3i/Zeiss two-photon upright microscope. The excitation wavelength was 950 nm. A 3 μL volume of a solution containing DsRed-AuBP (size 15 nm) bionanoprobes was injected into the ventral diencephalon area of a viable zebrafish brain.

An exciting aspect of our bionanoprobes is the prospect of bimodal imaging, that is, using multiple light emitting modalities to obtain measurements. Gold nanoparticles have unique local surface plasmon resonance properties, which allow them to either quench or enhance the fluoresce of the chromophores, depending on proximity [49,50,51], through increased excitation efficiency and radiative decay rates [50]. In Figure 6, we injected our bionanoprobes into live zebrafish brain and collected images, monitoring the 2PE fluorescence emission wavelengths at 950 nm (Figure 6A) and 850 nm (Figure 6B). Our results show that our bionanoprobe clusters not only emit fluorescent light at 950 nm, attributable to the bound DsRed protein, but also emit light at 850 nm, which is attributable to the plasmon resonance properties of gold nanoparticles. The peptide tag on the self-assembled fluorescent proteins on the gold nanoparticles may also contribute to stabilizing the distance between the chromophore region of the protein and the nanoparticle and improve the stability of the nanoprobe system for monitoring zinc ions. The combined measurement of emitted light at both wavelengths (Figure 6C) resulted in the easy identification of bionanoprobe clusters within the brain. Fluorescent image of nanoprobe clusters can be found at Figure S1.

**Figure 7:**
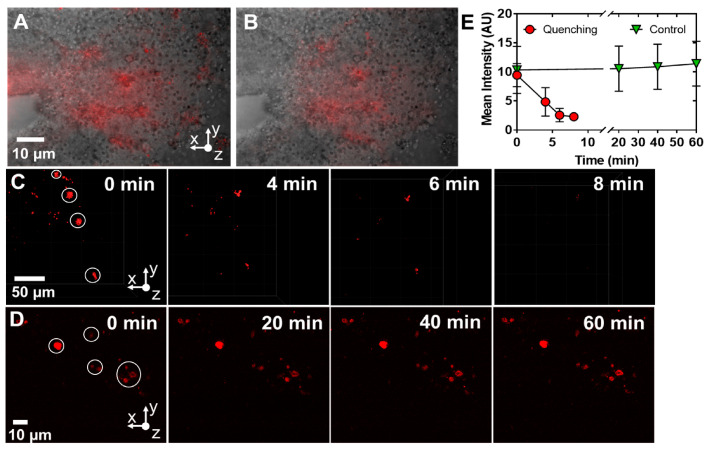
Exogenously applied Zn^2+^ rapidly quenches fluorescence. (**A**), A 15 µL volume of nanoparticles was injected into the telencephalon with a picospritzer (cartoon). (**B**), Injection of a 10 µL volume of Zn^2+^ at a concentration of 10 µM, resulting in a 41% decrease in total fluorescence within 2 s. Image obtained by overlaying fluorescence on brightfield images. (**C**), The brain was perfused with a solution of 100 nM ZnCl_2_ in aCSF. (**D**), No Zn^2+^ control. For all images, *x*-axis, caudal; *y*-axis, lateral; *z*-axis, ventral. (**E**), Graph of the average intensities of select regions within images in C and D. Regions analyzed are circled.

To determine the ability of free metal ions to quench fluorescence in living brain tissue, we injected a solution of ZnCl_2_ in aCSF into whole brains that had previously been injected with DsRed-BP AuNPs and imaged the nanoprobes with epifluorescence microscopy (Figure 7A). Upon the injection of ZnCl_2_ at a concentration of 10 μM, fluorescence decreased by 41% after 2 s (Figure 7B), indicating that the Zn^2+^ rapidly quenched the protein by association with either the metal-selective peptide sequence or another location on the DsRed protein. This result suggests that our probes can be used for time-resolved measurements of extracellular Zn^2+^ levels.

We further sought to determine if we could observe the fluorescence quenching of discrete bionanoprobe clusters. DsRed-BP-AuNPs probes were injected into the brain and the brain was perfused with ZnCl_2_ (100 nM) in aCSF while imaging with 2PE microscopy (Figure 7C). Clusters were present before and after the addition of Zn^2+^. However, over the course of 8 min, fluorescence almost completely disappeared, indicating rapid quenching. On the other hand, the fluorescence of bionanoprobes, injected without the perfusion of Zn^2+^, did not diminish over the course of 60 min (Figure 7D), demonstrating that the decrease in fluorescence occurred due to the quenching of the DsRed protein with Zn^2+^ rather than the photobleaching or degradation of the chromophore. The quantitation of the average fluorescence intensity also suggested that fluorescence is rapidly quenched by the application of Zn^2+^ to the superfusion buffer and that the controls remain stable over an extended time up to 60 min. When normalized against the controls at t = 0 min, the pooled quenched values obtained at 2, 4, and 6 min had significantly less fluorescence compared to the pooled control values at 20, 40, and 60 min (*p* = 0.0004, two-tailed *t*-test, n = four control and four quenched measurements).

## 3. Conclusions

In conclusion, engineered DsRed-AuBP-functionalized AuNP nanoprobes were readily quenched by Zn^2+^ in living brain tissue with good spatial and temporal resolution. They resisted photobleaching and degradation for at least an hour during imaging and remained stationary. Collectively, our results show that our bionanoprobes consisting of gold nanoparticles are well-suited for the measurement of Zn^2+^ at defined locations within the brain tissue.

Although our approach of measuring metals and other brain chemicals with functionalized nanoprobes is potentially useful, researchers should consider several factors prior to carrying out studies. The injection of bionanoprobes into tissues may be damaging; alternate methods of delivery may be advisable to minimize such damage. Other routes of entry into the brain may be through injection into the blood stream, oral ingestion, or aspirations. However, because we have not yet determined the blood brain permeability of our probes, the feasibility of these approaches is not known. An additional consideration is that the bionanoprobes may interfere with normal brain function and tend to accumulate in specific brain regions. Moreover, difficulties in quantitation and quenching by other ions represent other possible hindrances in the use of quenching nanoprobes. Nevertheless, our probes have shown excellent spectroscopic and mechanical stability. The confinement property of the bionanoprobes would make it possible to carry out site-specific studies at defined locations within the brain tissue. Monitoring the neuro-degenerative changes related to a Zn^2+^ imbalance across the zebrafish live brain tissue would contribute immensely to our current understanding of the complex molecular mechanisms underlying the pathogenesis of neurological disorders.

The current platform is amenable to being combined with other orthogonal methods in which nanoprobes could be tuned to target specific metal ions. These nanoprobes may also be used in bimodal applications in which fluorescence quenching is observed in addition to surface plasmon resonance from the gold portion of the nanoprobes. Our proposed bionanoprobes are versatile and can be incorporated with additional metal binding tags to engineered proteins for targeting different trace metal ions. The self-assembled system also means that the technology can be expanded to incorporate different nanoparticle-based systems. As a note, it is important to adhere to the guidelines for safety and ethical disposal when using nanomaterials.

In summary, this work offers a promising, targeted approach to address the growing demand for optical control for the in vivo sensing of key metal ions that is adaptable and can be easily implemented with other analytical methods in living tissue. As the multi-functionality of the nanoprobes expands, the novel molecular tools could impact our understanding of numerous neurological disease states, including Alzheimer’s disease, Parkinson’s disease, and Huntington’s disease.

## 4. Materials and Methods

Chemicals. The stock solution (10 µM) of ZnCl_2_ (CAS No. 7646-85-7, ≥98%, Sigma-Aldrich, St. Louis, MO, USA) was prepared by dissolving the appropriate analyte mass in modified artificial cerebrospinal fluid (aCSF). The aCSF consisted of 126 mM NaCl, 2.5 mM KCl, 2.4 mM CaCl_2_, 1.2 mM MgCl_2_, 25 mM NaHCO_3_, and 20 mM HEPES (all Sigma-Aldrich, St. Louis, MO, USA), and was adjusted to a pH of 7.4. Both stock solutions of ZnCl_2_ and aCSF were refrigerated when not in use. Ultrapure water (~18.2 MOhm-cm) was used to prepare all the aqueous solutions.

Animals. Adult *Danio rerio* (zebrafish, transgenic line *th2:gfp*) were housed in the Shankel Structural Biology Center at the University of Kansas, in 3L tanks (20 fish per 3L system rack tank) and connected to a recirculation filtration system. All the tanks were maintained under constant chemical, biological, and mechanical filtration conditions, as well as using a UV sterilizing unit to ensure adequate conditions. The following quality parameters of the reverse osmosis purified system water were controlled and adjusted using the multiparameter monitoring and control instrument 5200A (YSI, Yellow Springs, OH, USA): conductivity (~700 µS cm^−1^), pH (7.2), and temperature (28 °C). The fish were fed twice a day and maintained on a 14:10 light: dark cycle. The study was conducted according to the guidelines of the Declaration of Helsinki. All the protocols and procedures involving the zebrafish were approved by the Animal Care and Use Committee of the University of Kansas.

Euthanasia, brain perfusion, and extraction. All the zebrafish were euthanized by hypothermic shock followed by decapitation. Immediately following euthanasia, the whole brains were harvested using the previously described methods [52,53] and transferred to a perfusion chamber. The viability of the brains was ensured by the continuous flow of oxygenated and heated (28 °C) aCSF.

Protein-Peptide Construct Methods. The DsRed fluorescent protein conjugated with a gold-binding peptide (AuBP) and a maltose-binding protein (MBP) were expressed and purified from recombinant *E. coli* ER2507 cells following previously established protocols [41,42]. A single colony of the recombinant *E. coli* cells was grown sequentially into larger volumes (3 mL, 10 mL, and 400 mL) in LB-Miller media with 2% glucose and 100 µg/mL of ampicillin in an incubator (37 °C) with continuous shaking. The 400 mL solution of the recombinant *E. coli* cells was inoculated with IPTG (isopropyl β-D-1-thiogalactopyranoside) with a final concentration of 0.3 mM after an OD_600_ reading of 0.6 was reached. Once inoculated, the cells were incubated with continuous shaking overnight to give the cells time to express the fusion protein. The recombinant *E. coli* cells were then subsequently separated from the media through centrifugation at 4000× *g* for 30 min. The cell pellet was then resuspended in an amylose resin column buffer (20 mM Tris–HCl, 200 mM NaCl, 1 mM EDTA, pH 7.4) and lysed through sonication. The lysed cells were spun down through centrifugation at 10,000× *g* for 30 min. The supernatant was sterile filtered and loaded onto an amylose resin (New England Biolabs, NEB, Ipswich, MA, USA) column to purify the fusion protein using the maltose binding protein tag. The extraneous biomolecules were washed away with the column buffer, and the fusion protein was eluted using an elution buffer (column buffer + 10 mM of maltose). Using centrifugal filters with a 10 kDa (MilliporeSigma, Burlington, MO, USA) weight cut-off, the fusion protein was concentrated in Milli-Q sterile filtered water. Samples at the various steps of the purification process of the MBP-DsRed-AuBP were collected and verified using an SDS-PAGE gel. Then, the protein samples were transferred into a 1X cleavage buffer containing 20 mMTris-HCl, 100 mM NaCl, and 2 mM CaCl_2_ (pH 8.0) by ultrafiltration using the same centrifugal filter tube. A 40 μL volume of 1 mg/mL factor Xa (NEB, Ipswich, MA, USA) was added to 2.5 mg/mL of the fusion protein in a 1X cleavage buffer. The cleavage reaction was performed overnight at 16 °C. A buffer exchange was completed so the cleaved MBP and DsRed-AuBP was back in the column buffer and the mixture was added into a fresh amylose resin column. The cleaved MBP fragments bound to the resin and the flow through was collected as pure DsRed-AuBP. The fusion protein was again concentrated in Milli-Q sterile filtered water using centrifugal filters with a 10 kDa MWCO (MilliporeSigma, Burlington, MO, USA). Following cleavage, the purity of DsRed-AuBP was observed by SDS-PAGE.

AuNP Functionalization. The applicable safety precautions, including the use of personal protective equipment, a certified fume hood, and the proper disposal procedures were employed when handling the gold nanoparticles. Following a previously established protocol, stock, citrate-coated gold nanoparticles (AuNPs) were functionalized with the affinity-based DsRed-AuBP construct [39,40,42]. Briefly, stock AuNPs sized 15 and 50 nm (BBI Solutions, Portland, ME, USA) were provided at 47.8 and 56.9 µg/mL, respectively, and diluted in water to achieve a concentration of 10 µg/mL. To functionalize the AuNPs with the affinity peptide construct, the AuNPs were incubated with DsRed-AuBP and prepared to achieve a final concentration of 50 µM in water. The solution was allowed to incubate at room temperature (25 °C) for 2 h under gentle shaking conditions, protected from light to allow time for the peptide to bind to the gold surfaces. A wash step was completed to remove excess, unbound protein by pelleting the suspension via 30 min centrifugation at 17,000× *g* and 1100× *g* for 15 and 50 nm AuNPs, respectively, followed by carefully removing the supernatant. The pellet was reconstituted in either Milli-Q water or aCSF media to achieve a final functionalized AuNP concentration of 10 µg/mL in solution.

In vitro Fluorescence Quenching. To study the quenching effect of key metal ions on the DsRed-AuBP functionalized AuNPs, stock metal ion solutions were prepared. Magnesium sulfate heptahydrate (MgSO4; Fisher Chemical, Loughborough, UK), and zinc chloride (ZnCl2; Acros Organics) were prepared to achieve a working stock concentration of 6 mM in 10 mL of Milli-Q water and sterile filtered. In a 96-well plate, 100 µL of washed, DsRed-AuBP (ex/em 556/590 nm) functionalized AuNPs at a final concentration of 10 µg/mL were added into each well. Triplicate samples were prepared for each metal ion concentration tested. A 6X concentration of each metal ion was prepared from the working stock so that 20 µL could be added to each well immediately prior to the experimental measurements to achieve final, in-well ion concentrations of 0, 2, 4, 10, 25, 50, and 100 µM. Once the solutions were added, a fluorescence spectral scan was conducted to measure the quenching effect using a Cytation3 multi-modal plate reader. The excitation was kept at 525 nm to avoid interference with the emission spectral range from 556 to 680 nm. The immediate quenching of the fluorescence signal was noted with increasing ion concentrations.

Delivery of the nanoprobes. DsRed-BP-AuNP was injected into the telencephalon and diencephalon of living zebrafish whole brain with a micropipette linked to a Picospritzer III (Parker, Hollis, NH, USA). The micropipettes were fabricated from glass capillary tubes (1.2 mm D.D and 0.68 mm I.D, 4 in long; A-M System Inc., Carlsborg, WA, USA). The glass capillary tubes were pulled using a PE-22 heated coil puller (Narishige Int. USA, East Meadow, NY, USA) and cut to an external diameter of 20 µm.

Microscopy. Epifluorescence images were obtained with a Nikon E600Fn Epifluorescence microscope, using either a Plan Fluor 10X/0.30 NA air objective or a Fluor 40X/0.80 NA water immersion objective. The images were collected and analyzed with Metamorph software (Molecular Devices, San Jose, CA, USA).

Two-photon imaging was carried out on a customized 3i/Zeiss Axio Examiner.Z1 upright two-photon excitation microscope with a Plan-Apochromat 20X/1.0 NA water immersion objective. For the imaging of Au, DsRed-Au nanoparticles, and their combination with GFP-labeled neurons, excitation wavelengths of 850, 920, and 950 nm were used, respectively. 3D imaging was captured by resonant scanning and under the following parameters: resolution, 1024 × 1024 pixels; pixel size, 0.2441 µm; dwell time, 2 µs; bidirectional scanning, 1; voltage amplifier of the pockets, 0.5 V; and step size, 0.55 µm.

The activity of the nanoparticles (viability and movement) in the living brain were monitored by continuous imagining at 10 min intervals for periods lasting up to 2 h. The resulting 2-photon images were analyzed and post-processed using 3i SlideBook 6.0 (6.0.19 version), ImageJ software, and Adobe Photoshop.

## Data Availability

Not applicable.

## Supplementary Materials

The following supporting information can be downloaded at: https://www.mdpi.com/article/10.3390/molecules28052260/s1, Figure S1: Image of nanoprobe fluorescence from DsRed protein and gold nanoparticle fluorescence.

## Sample Availability

Samples of the proteins used are available from the authors.

## References

[1] Wang L., Yin Y.L., Liu X.Z., Shen P., Zheng Y.G., Lan X.R., Lu C.B., Wang J.Z. (2020). Current understanding of metal ions in the pathogenesis of Alzheimer’s disease. Transl. Neurodegener..

[2] Chen Z., Ai H.W. (2016). Single Fluorescent Protein-Based Indicators for Zinc Ion (Zn^2+^). Anal. Chem..

[3] Choi S., Hong D.K., Choi B.Y., Suh S.W. (2020). Zinc in the Brain: Friend or Foe?. Int. J. Mol. Sci..

[4] Howell G.A., Welch M.G., Frederickson C.J. (1984). Stimulation-induced uptake and release of zinc in hippocampal slices. Nature.

[5] Stewart G.R., Frederickson C.J., Howell G.A., Gage F.H. (1984). Cholinergic denervation-induced increase of chelatable zinc in mossy-fiber region of the hippocampal formation. Brain Res..

[6] Goldberg J.M., Lippard S.J. (2017). Challenges and Opportunities in Brain Bioinorganic Chemistry. Acc. Chem. Res..

[7] Frederickson C.J., Bush A.I. (2001). Synaptically released zinc: Physiological functions and pathological effects. Biometals.

[8] Frederickson C.J. (1989). Neurobiology of zinc and zinc-containing neurons. Int. Rev. Neurobiol..

[9] Slomianka L. (1992). Neurons of origin of zinc-containing pathways and the distribution of zinc-containing boutons in the hippocampal region of the rat. Neuroscience.

[10] Ketterman J.K., Li Y.V. (2008). Presynaptic evidence for zinc release at the mossy fiber synapse of rat hippocampus. J. Neurosci. Res..

[11] Takeda A., Fuke S., Tsutsumi W., Oku N. (2007). Negative modulation of presynaptic activity by zinc released from Schaffer collaterals. J. Neurosci. Res..

[12] Bagheri S., Squitti R., Haertle T., Siotto M., Saboury A.A. (2017). Role of Copper in the Onset of Alzheimer’s Disease Compared to Other Metals. Front. Aging Neurosci..

[13] Ward R.J., Dexter D.T., Crichton R.R. (2015). Neurodegenerative diseases and therapeutic strategies using iron chelators. J. Trace Elem. Med. Biol..

[14] Barnham K.J., Bush A.I. (2014). Biological metals and metal-targeting compounds in major neurodegenerative diseases. Chem. Soc. Rev..

[15] Qian X., Xu Z. (2015). Fluorescence imaging of metal ions implicated in diseases. Chem. Soc. Rev..

[16] Chowdhury S., Rooj B., Dutta A., Mandal U. (2018). Review on recent advances in metal ions sensing using different fluorescent probes. J. Fluoresc..

[17] Pratt E.P.S., Damon L.J., Anson K.J., Palmer A.E. (2021). Tools and techniques for illuminating the cell biology of zinc. Biochim. Biophys. Acta Mol. Cell Res..

[18] Qin Y., Dittmer P.J., Park J.G., Jansen K.B., Palmer A.E. (2011). Measuring steady-state and dynamic endoplasmic reticulum and Golgi Zn^2+^ with genetically encoded sensors. Proc. Natl. Acad. Sci. USA.

[19] Park J.G., Qin Y., Galati D.F., Palmer A.E. (2012). New sensors for quantitative measurement of mitochondrial Zn^2+^. ACS Chem. Biol..

[20] Fudge D.H., Black R., Son L., LeJeune K., Qin Y. (2018). Optical Recording of Zn^2+^ Dynamics in the Mitochondrial Matrix and Intermembrane Space with the GZnP2 Sensor. ACS Chem. Biol..

[21] Falcon-Perez J.M., Dell’Angelica E.C. (2007). Zinc transporter 2 (SLC30A2) can suppress the vesicular zinc defect of adaptor protein 3-depleted fibroblasts by promoting zinc accumulation in lysosomes. Exp. Cell Res..

[22] Kukic I., Lee J.K., Coblentz J., Kelleher S.L., Kiselyov K. (2013). Zinc-dependent lysosomal enlargement in TRPML1-deficient cells involves MTF-1 transcription factor and ZnT4 (Slc30a4) transporter. Biochem. J..

[23] Vinkenborg J.L., Nicolson T.J., Bellomo E.A., Koay M.S., Rutter G.A., Merkx M. (2009). Genetically encoded FRET sensors to monitor intracellular Zn^2+^ homeostasis. Nat. Methods.

[24] Yang Z., Loh K.Y., Chu Y.-T., Feng R., Satyavolu N.S.R., Xiong M., Nakamata Huynh S.M., Hwang K., Li L., Xing H. (2018). Optical control of metal ion probes in cells and zebrafish using highly selective DNAzymes conjugated to upconversion nanoparticles. J. Am. Chem. Soc..

[25] Peng J., Xu W., Teoh C.L., Han S., Kim B., Samanta A., Er J.C., Wang L., Yuan L., Liu X. (2015). High-efficiency in vitro and in vivo detection of Zn^2+^ by dye-assembled upconversion nanoparticles. J. Am. Chem. Soc..

[26] Carter K.P., Young A.M., Palmer A.E. (2014). Fluorescent Sensors for Measuring Metal Ions in Living Systems. Chem. Rev..

[27] Knecht M.R., Sethi M. (2009). Bio-inspired colorimetric detection of Hg^2+^ and Pb^2+^ heavy metal ions using Au nanoparticles. Anal. Bioanal. Chem..

[28] Zhang J., Cheng F., Li J., Zhu J.J., Lu Y. (2016). Fluorescent nanoprobes for sensing and imaging of metal ions: Recent advances and future perspectives. Nano Today.

[29] Lai J., Niu W., Luque R., Xu G. (2015). Solvothermal synthesis of metal nanocrystals and their applications. Nano Today.

[30] Li D., Ma Y., Duan H., Jiang F., Deng W., Ren X. (2018). Fluorescent/SERS dual-sensing and imaging of intracellular Zn^2+^. Anal. Chim. Acta.

[31] Li W., Nie Z., He K., Xu X., Li Y., Huang Y., Yao S. (2011). Simple, rapid and label-free colorimetric assay for Zn^2+^ based on unmodified gold nanoparticles and specific Zn^2+^ binding peptide. Chem. Commun..

[32] Promnimit S., Bera T., Baruah S., Dutta J. (2011). Chitosan capped colloidal gold nanoparticles for sensing zinc ions in water. Int. J. Nano Res..

[33] Tira D.S., Focsan M., Ulinici S., Maniu D., Astilean S. (2014). Rhodamine B-Coated Gold Nanoparticles as Effective “Turn-on” Fluorescent Sensors for Detection of Zinc II Ions in Water. Spectrosc. Lett..

[34] Wang S., Sun J., Gao F. (2015). A turn-on near-infrared fluorescent chemosensor for selective detection of lead ions based on a fluorophore-gold nanoparticle assembly. Analyst.

[35] Hnilova M., Karaca B.T., Park J., Jia C., Wilson B.R., Sarikaya M., Tamerler C. (2012). Fabrication of hierarchical hybrid structures using bio-enabled layer-by-layer self-assembly. Biotechnol. Bioeng..

[36] Hnilova M., Oren E.E., Seker U.O., Wilson B.R., Collino S., Evans J.S., Tamerler C., Sarikaya M. (2008). Effect of molecular conformations on the adsorption behavior of gold-binding peptides. Langmuir.

[37] Tamerler C., Duman M., Oren E.E., Gungormus M., Xiong X.R., Kacar T., Parviz B.A., Sarikaya M. (2006). Materials specificity and directed assembly of a gold-binding peptide. Small.

[38] Tamerler C., Oren E.E., Duman M., Venkatasubramanian E., Sarikaya M. (2006). Adsorption kinetics of an engineered gold binding peptide by surface plasmon resonance spectroscopy and a quartz crystal microbalance. Langmuir.

[39] Yuca E., Tamerler C. (2019). Self Assembled Recombinant Proteins on Metallic Nanoparticles As Bimodal Imaging Probes. JOM.

[40] Karaca B.T., Hnilova M., Tamerler C., Knecht M., Walsh T. (2014). Addressable biological functionalization of inorganics: Materials-selective fusion proteins in bio-nanotechnology. Bio-Inspired Nanotechnology.

[41] Karaca B.T., Meyer J., VanOosten S., Richter M., Tamerler C. (2014). Modular Peptide-Based Hybrid Nanoprobes for Bio-Imaging and Bio-Sensing. MRS Online Proc. Libr. Arch..

[42] Zhang S., Karaca B.T., VanOosten S.K., Yuca E., Mahalingam S., Edirisinghe M., Tamerler C. (2015). Coupling infusion and gyration for the nanoscale assembly of functional polymer nanofibers integrated with genetically engineered proteins. Macromol. Rapid Commun..

[43] Ko S.-K., Chen X., Yoon J., Shin I. (2011). Zebrafish as a good vertebrate model for molecular imaging using fluorescent probes. Chem. Soc. Rev..

[44] Kalueff A.V., Stewart A.M., Gerlai R. (2014). Zebrafish as an emerging model for studying complex brain disorders. Trends Pharmacol. Sci..

[45] Li W., Fang B., Jin M., Tian Y. (2017). Two-photon ratiometric fluorescence probe with enhanced absorption cross section for imaging and biosensing of zinc ions in hippocampal tissue and zebrafish. Anal. Chem..

[46] Kim E.H., Chin G., Rong G., Poskanzer K.E., Clark H.A. (2018). Optical Probes for Neurobiological Sensing and Imaging. Acc. Chem. Res..

[47] Iversen T.G., Skotland T., Sandvig K. (2011). Endocytosis and intracellular transport of nanoparticles: Present knowledge and need for future studies. Nano Today.

[48] Kiselyov K., Colletti G.A., Terwilliger A., Ketchum K., Lyons C.W.P., Quinn J., Muallem S. (2011). TRPML: Transporters of metals in lysosomes essential for cell survival?. Cell Calcium..

[49] Fan C., Wang S., Hong J.W., Bazan G.C., Plaxco K.W., Heeger A.J. (2003). Beyond superquenching: Hyper-efficient energy transfer from conjugated polymers to gold nanoparticles. Proc. Natl. Acad. Sci. USA.

[50] Li S., Zhang T., Zhu Z., Gao N., Xu Q.-H. (2016). Lighting up the gold nanoparticles quenched fluorescence by silver nanoparticles: A separation distance study. RSC Adv..

[51] Swierczewska M., Lee S., Chen X. (2011). The design and application of fluorophore-gold nanoparticle activatable probes. Phys. Chem. Chem. Phys..

[52] Field T.M., Shin M., Stucky C.S., Loomis J., Johnson M.A. (2018). Electrochemical Measurement of Dopamine Release and Uptake in Zebrafish Following Treatment with Carboplatin. Chemphyschem.

[53] Shin M., Field T.M., Stucky C.S., Furgurson M.N., Johnson M.A. (2017). Ex Vivo Measurement of Electrically Evoked Dopamine Release in Zebrafish Whole Brain. ACS Chem. Neurosci..

