# Supplementary material for: Spatiotemporal Imaging of Zinc Ions in Zebrafish Live Brain Tissue Enabled by Fluorescent Bionanoprobes
Source: Molecules. 2023 Feb 28;28(5):2260. doi: 10.3390/molecules28052260 (PMC10005619; doi:10.3390/molecules28052260)
Supplement: Supplementary file 1 [file molecules-28-02260-s001.zip › molecules-2137774-supplementary.pdf]

Supplementary Material

Spatiotemporal Imaging of Zinc Ions in Zebrafish Live Brain Tissue Enabled by Fluorescent Bionanoprobes

Romana Jarosova [1,2], Sarah K. Woolfolk [3, 4], Noraida Martinez-Rivera [5], Matthew W. Jaeschke [3, 6], Eduardo Rosa-Molinar [5, 7], Candan Tamerler*[3, 4, 6], and Michael A. Johnson*[1]

[1] Department of Chemistry and R.N. Adams Institute for Bioanalytical Chemistry, University of Kansas, Lawrence, Kansas 66045, USA

[2] UNESCO Laboratory of Environmental Electrochemistry, Department of Analytical Chemistry, Charles University, Prague 2 12843, Czech Republic

[3] Institute for Bioengineering Research, University of Kansas, Lawrence, KS,66045, USA

[4] Bioengineering Program, University of Kansas, Lawrence, KS 66045, USA

[5] Microscopy and Analytical Imaging Research Resource Core Laboratory, University of Kansas, Lawrence, KS 66045, USA

[6] Department of Mechanical Engineering, University of Kansas, Lawrence, Kansas 66045, USA

[7] Department of Pharmacology & Toxicology, University of Kansas, Lawrence, KS 66045, USA

* correspondance: johnsonm@ku.edu (MAJ), ctamerler@ku.edu (CT).

1. Image of nanoprobe fluorescence from DsRed protein and gold nanoparticle fluorescence.

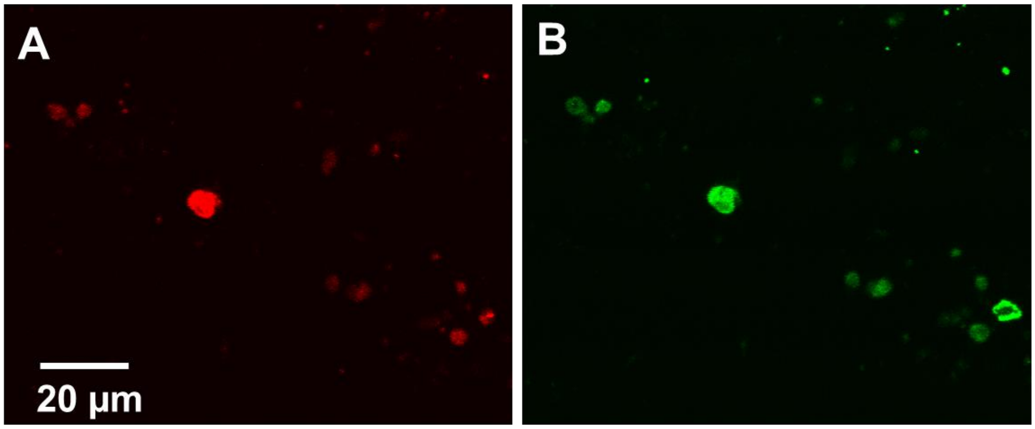

Figure S1. Fluorescent image of nanoprobe clusters. (A) Fluorescence of DsRed protein on nanoprobes measured at 950 nm. (B) Fluorescence of gold portion of nanoprobe measured at 850 nm. Images collected on 3i/Zeiss Two-Photon Upright Microscope. Excitation wavelength was 950 nm.
